# Thermal Conversion of Unsolvated Mg(B_3_H_8_)_2_ to BH_4_^–^ in the Presence of MgH_2_

**DOI:** 10.1021/acsaem.1c00159

**Published:** 2021-04-02

**Authors:** Angelina Gigante, Noemi Leick, Andrew S. Lipton, Ba Tran, Nicholas A. Strange, Mark Bowden, Madison B. Martinez, Romain Moury, Thomas Gennett, Hans Hagemann, Tom S. Autrey

**Affiliations:** † Département de Chimie Physique, Université de Genève, 30, quai E. Ansermet, 1211 Geneva 4, Switzerland; ‡ National Renewable Energy Laboratory, 15013 Denver W Pkway, Golden, Colorado 80401, United States; § Environmental Molecular Division, Earth and Biological Sciences Directorate, Pacific Northwest National Laboratory, Richland, Washington 99354, United States; ∥ Physical Sciences Division, Physical and Computational Sciences Directorate, Pacific Northwest National Laboratory, Richland, Washington 99354, United States; ⊥ SLAC National Accelerator Laboratory, 2575 Sand Hill Road, Menlo Park, California 94025, United States; # Institut des Molécules et des Matériaux du Mans, UMR 6283 CNRS, Le Mans Université, Avenue Olivier Messiaen, 72085 Le Mans Cedex 9, France; ∇ Chemistry Department, Colorado School of Mines, 1012 14th Street, Golden, Colorado 80401, United States

**Keywords:** unsolvated magnesium octahydrotriborate, magnesium hydride, nuclear magnetic resonance, thermal conversion, renewable energy

## Abstract

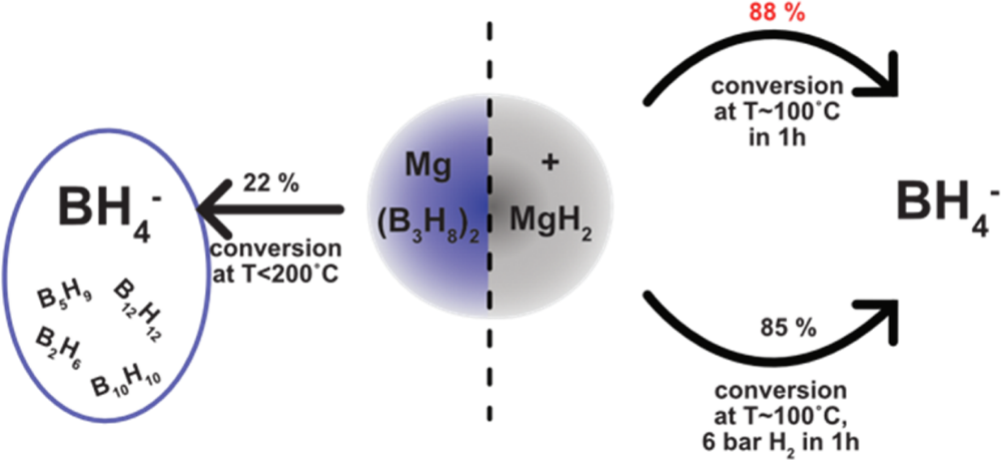

In the search for energy storage materials, metal octahydrotriborates, M(B_3_H_8_)*_n_*, *n* = 1 and 2, are promising candidates for applications such as stationary hydrogen storage and all-solid-state batteries. Therefore, we studied the thermal conversion of unsolvated Mg(B_3_H_8_)_2_ to BH_4_^–^ as-synthesized and in the presence of MgH_2_. The conversion of our unsolvated Mg(B_3_H_8_)_2_ starts at ∼100 °C and yields ∼22 wt % of BH_4_^–^ along with the formation of (closo-hydro)borates and volatile boranes. This loss of boron (B) is a sign of poor cyclability of the system. However, the addition of activated MgH_2_ to unsolvated Mg(B_3_H_8_)_2_ drastically increases the thermal conversion to 85–88 wt % of BH_4_^–^ while simultaneously decreasing the amounts of B-losses. Our results strongly indicate that the presence of activated MgH_2_ substantially decreases the formation of (closo-hydro)borates and provides the necessary H_2_ for the B_3_H_8_-to-BH_4_ conversion. This is the first report of a metal octahydrotriborate system to selectively convert to BH_4_^–^ under moderate conditions of temperature (200 °C) in less than 1 h, making the MgB_3_H_8_-MgH_2_ system very promising for energy storage applications.

## Introduction

To reach a widespread implementation and integration of renewable energy systems, the development of new energy storage materials is crucial to mitigate the intermittency and unpredictability of renewable energy sources, such as wind or sun, thereby leading to a more reliable power grid. Boron hydrogen (B*_x_*H*_y_*) compounds are a unique class of materials due to their three-center two-electron (3c-2e) bonding providing a variety of materials promising for energy storage applications.^1−5^ Recently, this class of compounds has been demonstrated to be promising candidates as solid-state electrolytes for all-solid-state batteries.^6−10^ For example, KB_3_H_8_ was reported to reach a maximum conductivity of 3.4 × 10^–7^ S cm^–1^ at 150 °C,^11^ and that of unsolvated Mg(B_3_H_8_)_2_ reached 1.4 × 10^–4^ S cm^–1^ at 80 °C.^12^ Compounds based on the B_3_H_8_^–^ anion have also been studied as nonflammable hydrogen carriers at room temperature in order to address the flammability issues related to the transport of gasoline. Borohydride-based ionic liquids, such as C(NH_2_)_3_B_3_H_8_ and Li(NH_3_)B_3_H_8_, are considered due to their melting point less than −10 °C and their liquid nature at room temperature. While C(NH_2_)_3_B_3_H_8_ releases ∼7 mol % of H_2_ at 83 °C, Li(NH_3_)B_3_H_8_ releases ∼95 mol % of H_2_ at 133 °C along with the liberation of minor B_2_H_6_ and B_5_H_9_ amounts.^13,14^ By adding metal chlorides, MCl*_x_* (*x* = 2–4, M = Al, Zn, Zr), the H_2_ evolution of the Li(NH_3_)B_3_H_8_ was improved to ∼97, 98, and 96 mol % at 109–119 °C.^14^ B_3_H_8_^–^ compounds, such as NaB_3_H_8_ (13 wt % H) and KB_3_H_8_ (5 wt % H), have been also recently studied for long-term solid-state hydrogen storage.^11,15−17^ In the case of NaB_3_H_8_, it has a high solubility in water (74 wt %) at room temperature; therefore, possibilities exist for it to be used as a liquid hydrogen carrier in solution. Huang et al. found that the use of CoCl_2_ can act as a catalyst to enhance the hydrogen evolution up to ∼7 wt % in the case of NaB_3_H_8_.^18^ Compounds based on B_3_H_8_^–^ are also potential materials in preparing metal borides (MB*_x_*, *x* = 1, 2) with chemical vapor deposition methods. For instance, Cr(B_3_H_8_)_2_, Mg(B_3_H_8_)_2_(Et_2_O)_2_, and Mg(B_3_H_8_)_2_(MeO)_2_ were used to synthesize CrB_2_ and MgB_2_. However, the evolution of pentaborane (B_5_H_9_) from heating these precursors affects the purity of the deposited metal boride films.^19,20^

Mg(B_3_H_8_)_2_ was first observed experimentally as a dehydrogenation intermediate of magnesium borohydride, Mg(BH_4_)_2_, at 200 °C in vacuo after 5 weeks.^21^ This result pioneered research initiatives to determine the reversible re-hydrogenation of Mg(B_3_H_8_)_2_ to Mg(BH_4_)_2_ according to the following reaction: Mg(B_3_H_8_)_2_ + MgH_2_ + H_2_ → Mg(BH_4_)_2_. To investigate the reversible hydrogenation of Mg(BH_4_)_2_ from Mg(B_3_H_8_)_2_, Chong et al. used tetrahydrofuran (THF) and studied the hydrogenation of solvated Mg(B_3_H_8_)_2_·2THF.^22^ The authors found that the system Mg(B_3_H_8_)_2_·2THF-MgH_2_ yielded a 9 mol %-conversion to BH_4_^–^ at 200 °C under 50 bar of H_2_. However, the role of MgH_2_ and THF in the hydrogenation of Mg(B_3_H_8_)_2_·2THF-MgH_2_ was not clarified. The finding boosted the development of synthetic procedures for B_3_H_8_^–^ compounds and the study of its properties. Initially, synthetic routes of M(B_3_H_8_)*_n_*, *n* = 1 and 2, involved the use of toxic chemicals (e.g., Hg, B_2_H_6_, B_4_H_10_, and BF_3_O(C_2_H_5_)_2_),^23−30^ while the more recent synthetic strategies reported are solvent-free for M(B_3_H_8_)*_n_* with *n* = 1 and 2 and M = Mg, Li, Na, K, Rb, and Cs.^12,31−34^ Theoretical calculations based on prototype electrostatic ground state search method and density functional theory suggested that unsolvated Mg(B_3_H_8_)_2_ had an unfavorable enthalpy of reaction to be a decomposition product of Mg(BH_4_)_2_ since it was estimated to be +180 kJ/mol H_2_ for the following reaction: Mg(BH_4_)_2_ → Mg(B_3_H_8_)_2_ + MgH_2_ + H_2_.^35^ However, the authors used a computed crystal structure of Mg(B_3_H_8_)_2_ due to the lack of experimental data at that time. Recently, Moury et al. have revealed that unsolvated Mg(B_3_H_8_)_2_ was mainly amorphous.^12^ This finding will open more opportunities in studying unsolvated Mg(B_3_H_8_)_2_ and its chemical and physical properties in view of energy storage materials.

One of the greatest challenges encountered by these materials is the ability to de-/rehydrogenate because (i) they lose boron in the form of gas-phase boranes in their thermal decomposition, (ii) they are not selective in their decomposition products as species like B_10_H_10_^2–^ are produced, and (iii) they form solid-state species regarded as thermodynamic sinks, such as B_12_H_12_^2–^. For example, the thermal decomposition of KB_3_H_8_ occurs in the temperature range of 150–250 °C with a mass loss of 11 wt %, with the generation of several gaseous by-products such as B_2_H_6_, B_5_H_9_, and B_6_H_10_ and solid products B_12_H_12_^2–^, B_10_H_10_^2–^, and BH_4_^–^.^17^ To date, the reported hydrogen cycling capacity of Mg(B_3_H_8_)_2_ formed from dehydrogenation of Mg(BH_4_)_2_ is ∼2.5 wt % due to the formation these by-products.^21,36^ In solid-state batteries, it has recently been shown that the cation conductivity is enabled by the high mobility of the B_3_H_8_^–^ anion.^12,37^ During discharging of the battery, the B_3_H_8_^–^ forms diborane and pentaborane, preventing sufficient discharge/charge cycles to be considered in an application.

With the intent of facilitating the hydrogenation of metal octahydrotriborates, KH was added to KB_3_H_8_,^11^ and a conversion from KB_3_H_8_^–^-KH to KBH_4_ was observed at 150 °C under 382 bar of H_2_. Room-temperature solid-state nuclear magnetic resonance (NMR) of the solid hydrogenated mixture revealed a conversion of 36 mol % into BH_4_^–^ along with the formation of 26 mol % B_12_H_12_^2–^, 4.5 mol % B_10_H_10_^2–^, 14 mol % B_9_H_9_^2–^, and an overall quantity of borates, BO*_x_*, *x* = 2–4, estimated at 13 mol %. Furthermore, it was reported that metal hydrides can react with B_2_H_6_ in solution to yield BH_4_^–^ in ether solvents such as diglyme, which could facilitate hydrogenation. Specifically, Batha et al. observed the reaction of MgH_2_ with B_2_H_6_ to give Mg(BH_4_)_2_ in the temperature range of 25–90 °C, and Brown and Tierney obtained LiBH_4_ by reacting LiH with B_2_H_6_.^38,39^ Additionally, the synthesis of LiBH_4_ and Mg(BH_4_)_2_ was demonstrated by reacting LiH and MgH_2_ with B_2_H_6_ at 120 °C.^40,41^

In this work, our approach adopted the addition of MgH_2_ to unsolvated Mg(B_3_H_8_)_2_ as a strategy to substantially retain the boron species of unsolvated Mg(B_3_H_8_)_2_ to address the cyclability issues of these materials. Simultaneously, this work shows that the Mg(B_3_H_8_)_2_-MgH_2_ system yields a very high B_3_H_8_^–^ to BH_4_^–^ conversion of 85–88 wt % at 200 °C in 1 h, without the supply of excess hydrogen gas. This result is currently the highest conversion to BH_4_^–^ observed in the hydrogenation of all metal-octohydrotriboranes compounds, and it is the first example reported of the conversion to BH_4_^–^, without the supply of additional hydrogen.

The use of MgH_2_ as a reactive hydride composite has been reported earlier.^42,43^ In these systems, two hydrides are combined with each other in order to add an exothermic reaction to the endothermic hydrogen desorption reaction to form a new compound, thereby lowering the overall reaction enthalpy to release hydrogen. In the case of MgH_2_ and LiBH_4_, MgB_2_ (Δ_f_*H*° = −91.96 kJ/mol) is formed.^44^ In this work, our approach adopted the addition of MgH_2_ to unsolvated Mg(B_3_H_8_)_2_ as a strategy to substantially retain the boron species of unsolvated Mg(B_3_H_8_)_2_ to address the cyclability issues of these materials. The new and pertinent result presented here is that MgH_2_, which has been added to keep a constant Mg/B ratio as in Mg(BH_4_)_2_, does not catalyze the further decomposition of Mg(B_3_H_8_)_2_ but rather favors the rehydrogenation reaction and prevents the release of toxic boranes. In this case, the thermodynamic driving force is the formation of Mg(BH_4_)_2_, which has a formation enthalpy of Δ_f_*H*° = −208 kJ/mol.^45^ This raises the possibility that excess MgH_2_ may also favor the rehydrogenation of other compounds of the form MgB*_x_*H*_y_*.

Because we study the thermal decomposition and the products formed upon the decomposition process, the terms “decomposition” and “conversion” will be used interchangeably in this publication. To demonstrate these results, we first characterize the de-/rehydrogenation products of unsolvated Mg(B_3_H_8_)_2_, with a gravimetric capacity of 15 wt % developed from a novel synthesis involving tetra-alkylammonium salts of B_3_H_8_^–^.^12^ The de-/rehydrogenation of our unsolvated Mg(B_3_H_8_)_2_ in the presence of MgH_2_ is studied with and without pressurizing the system to 6 bar of H_2_. The dominant characterization techniques used in this work are ^11^B solid-state magic angle spinning (MAS) NMR to determine the B-species present in the samples, temperature programmed desorption coupled with mass spectrometry (TPD-MS) to follow the evolution of volatile borane species, and X-ray absorption near edge structure (XANES) measurements for the identification and quantification of Mg- and B-species formed during the dehydrogenation.

## Experimental Details

All preparations of materials/samples for analysis were performed in an argon (Ar) filled glovebox (H_2_O < 0.1 ppm, O_2_ < 0.1 ppm). Three stainless steel balls with 10 mm diameters (ball-to-reactant mass ratio ∼40) were placed in a 65 mL airtight milling vessel for the synthesis of unsolvated Mg(B_3_H_8_)_2_, Mg(B_3_H_8_)_2_-MgH_2_, and the activation of MgH_2_ used in ^11^B Solid-state MAS NMR and XANES experiments. Five stainless steel balls with 10 mm diameters (ball-to-reactant mass ratio ∼40) were placed in a 20 mL airtight milling vessel for the synthesis of unsolvated Mg(B_3_H_8_)_2_, Mg(B_3_H_8_)_2_-MgH_2_, and the activation of MgH_2_ for the TPD-MS experiments.

### Sample Synthesis

NaB_3_H_8_ was synthesized according to the procedure developed by Moury et al.^33^ The details of the synthesis of NaB_3_H_8_ are reported in the Supporting Information. The as-synthesized NaB_3_H_8_ was mechanochemically converted into unsolvated Mg(B_3_H_8_)_2_ using the corresponding bromide salt as described by the following reaction: 2NaB_3_H_8_+ MgBr_2_ → Mg(B_3_H_8_)_2_ + 2NaBr.

Unsolvated Mg(B_3_H_8_)_2_ was synthesized by milling 0.2020 g of NaB_3_H_8_ with 0.3125 g of MgBr_2_ in air-tight milling vessels. The vessels were filled in an Ar glovebox and then milled for 1 h at 200 rpm (15 min milling and 5 min break). Note about the compound unsolvated Mg(B_3_H_8_)_2_ is highly pyrophoric and it must be kept away from water. It is highly recommended to store it in an inert glovebox environment.

The activation of MgH_2_ was achieved by ball milling. Before the synthesis of Mg(B_3_H_8_)_2_-MgH_2_, 0.2000 g of commercial MgH_2_ was introduced in an airtight vessel and milled for 2 h at 450 rpm (5 min milling, 1 min break). The activated MgH_2_ was stored in the glovebox.

Mg(B_3_H_8_)_2_-4MgH_2_ was prepared by mixing 0.0400 g of Mg(B_3_H_8_)_2_ with 0.0135 g of MgH_2_ in an airtight milling vessel according to the following parameters: 35 min at 300 rpm (5 min milling and 1 min break). 0.0400 g of Mg(B_3_H_8_)_2_ included the reaction product NaBr and residual MgBr_2_ from the synthesis. The synthetized product was stored in the glovebox. Throughout this manuscript, we will use the nomenclature “Mg(B_3_H_8_)_2_-MgH_2_” to refer to the material obtained by a physical mixture of Mg(B_3_H_8_)_2_ and 4 weight equivalents of MgH_2_. The details about the cleaning of the jars after the activation of MgH_2_ and the synthesis of Mg(B_3_H_8_)_2_-MgH_2_ are provided in Section S1.

### Material Characterization

#### ^11^B Solid-State MAS NMR

^11^B solid-state MAS NMR experiments were performed in an Oxford Instruments 11.7 T widebore magnet, an Agilent VNMRS spectrometer, and a homebuilt 5 mm HX probe (ceramic MAS housing from Revolution NMR) in the EMSL user facility at Pacific Northwest National Laboratory, USA. ^1^H and ^11^B signals were referred to tetramethylsilane at 0 ppm and boric acid 1 M at 20 ppm and measured at 500.1 and 160.4 MHz, respectively. The RF fields for direct polarization were a π/20 pulse of 0.26 μs for ^11^B and a ^1^H decoupling field of 42 kHz utilizing a SPINAL-16 scheme.^46^

The rotors are 5 mm cavern-style zirconia sleeves (Revolution NMR) that have been modified to accommodate a double O-ring Vespel bushing to seal the sample up to 225 bar at 250 °C that were previously described.^47^ The samples were packed into the rotor within the glovebox and sealed with the Vespel bushing and needle valve with Viton O-rings. A picture of the rotor is provided in Figure S1. The rotor was spun at 5 kHz at room temperature and heated to achieve a sample temperature of 100, 185, and 200 °C with a heating ramp of 10 °C/min (calibrated with a rotor of lead nitrate^48^ spun at 5 kHz). A spectrum was recorded at each temperature as well as after cooling the sample to 25 °C. For the hydrogenation experiment, the sample was pressurized with H_2_ utilizing a one-way valve end cap.^47,49^

The rotor was loaded in the glovebox and sealed with a one-way valve. The sealed rotor was then placed in a pressurizing chamber. The chamber was evacuated to ≈1 × 10^–3^ bar to remove any air and was then repressurized with H_2_ at 6 bar for 30 min to equilibrate. The presence of H_2_ was verified by ^1^H solid-state MAS NMR by comparing the ^1^H NMR spectrum before and after introducing H_2_. These results as well as experimental details related to liquid NMR can be found in Figures S2 and S3 and Section S2. The quantification procedure of the species measured by NMR is laid out in the Supporting Information, and the accuracy of the values obtained is within 5 wt %.

#### TPD-MS

The samples studied for this work were analyzed on a calibrated, custom-built TPD-MS system equipped with the quadrupole mass spectrometer (QMS) Stanford Research Systems RGA 100, with *m*/*z* = 1–100 amu, sampling rate of 2–4 s, and 70 eV ionization energy. The samples were heated at a rate of 10 °C/min from 26 to 400 °C utilizing a Digi-Sense temperature controller. The quantity of material was adjusted to stay within the calibrated mass spectrometer’s linear response region. In a typical analysis, 1–2 mg of sample was placed inside a platinum (Pt) foil packet to ensure homogeneous heating of the entire sample during the dehydrogenation process. The sample was then inserted into a quartz tube mounted to the TPD system, as described elsewhere.^50^ Care was taken with the ramp rates to ensure that the pumping rate of the system was not altered by the release of hydrogen. All experimental parameters were controlled via a LabView interface with the RGA, heating system, and pressure gauges. Typical initial pressures before heating the sample were at 10^–8^ torr, with a flat baseline, i.e., no water, hydrogen, or air signals above background.

The signal for H_2_ (*m*/*z* = 2) was calibrated on the system using the well-known H_2_ capacity of TiH_2_ (Alfa Aesar) (4.0 wt % H_2_). Three different sample sizes were tested, and the capacity value extracted from TPD has an accuracy of ±10%. The TiH_2_ calibration samples were heated at a rate of 10 °C/min from 25 to 700 °C.

#### XANES

Following the same recipe as described above, a fresh batch of samples was specifically prepared a few days in advance of the XANES measurements. All information related to synthesis and characterization of the second batch is presented in Section S1.5 as well as a comparison of the two sample batches discussed in this work. XANES measurements were acquired in a UHV chamber at Stanford Synchrotron Radiation Light source (SSRL) beam line 10–1. Powders were adhered to carbon tape and mounted to an aluminum sample stick oriented at 55° relative to the beam path. X-ray absorption spectra were obtained in the near edge region (−15 to +100 eV) over the Mg K (1 s) edges. Signals were acquired with simultaneous total electron yield (TEY) and total fluorescence yield (TFY). In this type of measurement, TEY is sensitive to the near-surface phenomena while TFY provides a signal more representative of the bulk-phase. Edge steps were normalized using Athena, and edge positions were located by performing a Gaussian peak fit to the first numerical derivative of the normalized data. Linear combination fits to the data were also performed in Athena using the starting Mg(B_3_H_8_)_2_ and MgH_2_ materials and a reference Mg(BH_4_)_2_ sample as standard.

## Results and Discussion

### Characterization of the Unsolvated Mg(B_3_H_8_)_2_ and the Mg(B_3_H_8_)_2_-MgH_2_ Mixture

**Figure 1:**
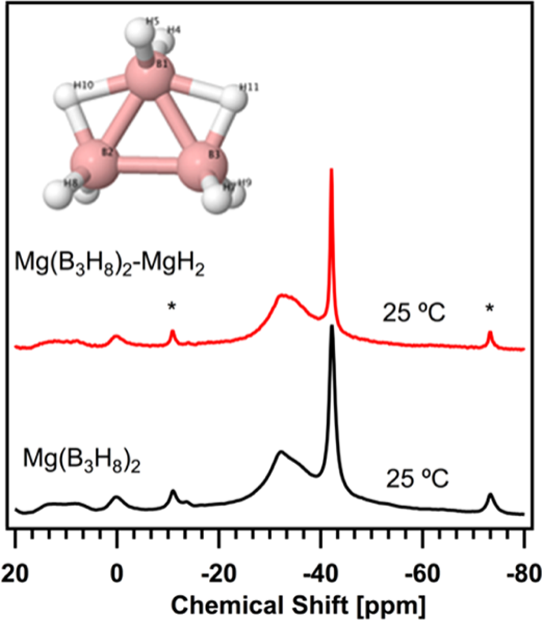
^11^B solid-state MAS NMR spectra of the unsolvated Mg(B_3_H_8_)_2_ (black spectrum) and the ball-milled Mg(B_3_H_8_)_2_-MgH_2_ (red spectrum) at 11.7 T, 5 kHz MAS, and 25 °C. The asterisks indicate the locations of spinning side bands at −11.0 and −73.0 ppm. The inset depicts the structure of the B_3_H_8_^–^ ion, reproduced from ref (56).

The unsolvated Mg(B_3_H_8_)_2_ was prepared according to the procedure recently reported by Moury et al.^12^ The compound was analyzed by ^11^B solid-state MAS NMR at 25 °C, and the chemical shifts are shown in Figure 1. The peak assignments and quantification from the integrated intensities are reported in Table 1. The ^11^B solid-state MAS NMR spectrum shows the spinning side bands centered at −11.0 and −73.0 ppm as indicated by asterisks in Figure 1. The peaks at −27.0, −34.0, and −31.6 ppm reveal the presence of the B_3_H_8_^–^ anion,^51^ with a content of 78 wt %. This broad B_3_H_8_^–^ signal can be explained by (i) the fluxional behavior of the protons due to the rapid exchange of the terminal hydrogens with the bridging hydrogens^52−55^ as well as by (ii) the amorphous nature of unsolvated Mg(B_3_H_8_)_2_, recently observed by XRD performed at 25 °C.^12^

**Table 1:** Identification of the Chemical Shifts and Quantification of the Chemical Compositions of the Unsolvated Mg(B_3_H_8_)_2_ and Ball-Milled Mg(B_3_H_8_)_2_-MgH_2_ Obtained by ^11^B Solid-State MAS NMR at 11.7 T, 5 kHz MAS, and 25 °C

sample	B_3_H_8_^–^ [ppm]	BH_4_^–^ [ppm]	BO*_x_* [ppm]
Mg(B_3_H_8_)_2_	–27.0, −34.0, −31.6	–42.2, −42.4	–0.1, 8.0, 13.0
Mg(B_3_H_8_)_2_-MgH_2_	–29.0, −27.7	–42.1, −39.7	–0.2, 8.0, 14.0

sample	B_3_H_8_^–^ [wt %]	BH_4_^–^ [wt %]	BO*_x_* [wt %]
Mg(B_3_H_8_)_2_	78	12	10
Mg(B_3_H_8_)_2_-MgH_2_	82	9	9

Additionally, the signals at −42.2 and −42.4 ppm in Figure 1 signify the presence of BH_4_^–^ (12 wt %) in the starting material along with borate species (BO*_x_*).^51,57−59^ The amount of BH_4_^–^ in the starting material will constitute a baseline quantity for the thermal conversion study. Since the synthesis of Mg(B_3_H_8_)_2_ involves NaB_3_H_8_, the ^11^B solid-state MAS NMR was not able to distinguish Mg(BH_4_)_2_ from NaBH_4_; therefore, we performed ^11^B solid-state MAS NMR of the as-synthesized NaB_3_H_8_ at 25 °C, shown in Figure S4, in the Section S3 revealing the presence of both B_3_H_8_^–^ (80 wt %) and BH_4_^–^ (15 wt %) as summarized in Table S1. Thus, the −42.2 and −42.4 ppm peaks shown in Figure 1 can originate from either Mg(BH_4_)_2_ or NaBH_4_.

The Mg(B_3_H_8_)_2_-MgH_2_ system was obtained by mixing Mg(B_3_H_8_)_2_ with 4 weight-equivalent activated MgH_2_ by ball milling. The ^11^B solid-state MAS NMR spectrum of the obtained Mg(B_3_H_8_)_2_-MgH_2_ mixture at 25 °C is shown in Figure 1 (red spectrum). The contents of B_3_H_8_^–^ (−29.0, −27.7 ppm) and BH_4_^–^ (−42.1, −39.7 ppm) were estimated to be 82 and 9 wt % respectively, as summarized in Table 1. The mechanochemical synthesis of unsolvated Mg(B_3_H_8_)_2_ relies on the metathesis reaction between NaB_3_H_8_ and MgBr_2_ leading to the formation of NaBr as a reaction by-product, as shown in the Experimental Details. The presence of residual MgBr_2_ in the diffraction pattern of Mg(B_3_H_8_)_2_-MgH_2_ shown in Figure S5 indicates that this reaction was not entirely completed in this case. The presence of NaBr and MgBr_2_ can potentially play a role, albeit minor, in the decomposition reaction of Mg(B_3_H_8_)_2_-MgH_2_. It has been shown that the ionic radii of Br^–^ and BH_4_^–^ are of similar size,^45^ making it the formation of mixed salts, such as Mg(BH_4_)_2–*x*_Br*_x_*, possible during heating of Mg(B_3_H_8_)_2_ in the presence of NaBr or MgBr_2_. Furthermore, the ionic substitution of halide for BH_4_ was shown to modify the stability of metal borohydrides, specifically of NaBH_4_ and LiBH_4_.^60,61^ However, with our experimental data available, it is very difficult to appreciate the extent of this mixed salt formation.

**Figure 2:**
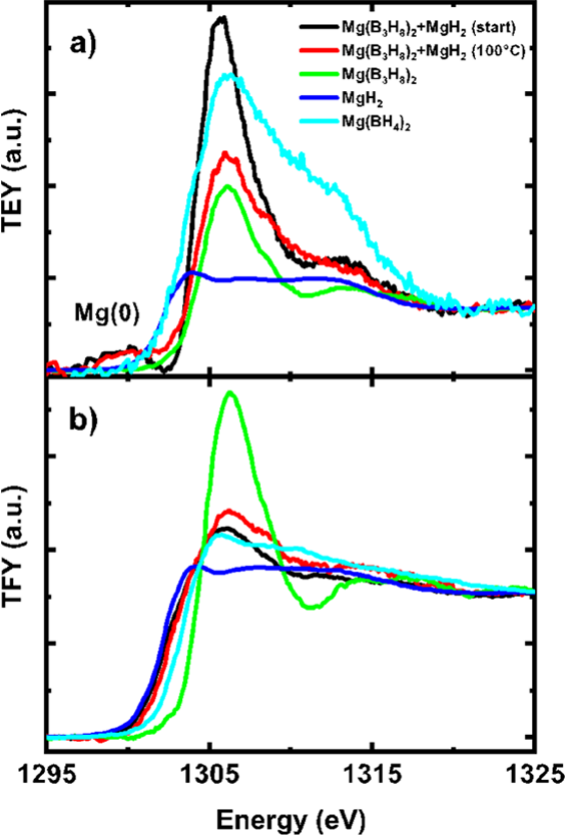
XANES spectra at the Mg K-edge where panel (a) depicts the TEY signal, which is surface-sensitive, and panel (b) depicts the TFY representative of the bulk contribution of the as-synthesized unsolvated Mg(B_3_H_8_)_2_-MgH_2_ sample compared to the heated Mg(B_3_H_8_)_2_-MgH_2_ system, the unsolvated Mg(B_3_H_8_)_2_, activated MgH_2_ (blue), and Mg(BH_4_)_2_ (cyan).

The XANES spectra displayed in Figure 2 show the surface-sensitive total electron yield (TEY) and the fluorescence yield (TFY) signals from the sample, recorded at the Mg K-edge at 25 °C. The synthesis, characterization, and comparison of this second batch with the main material investigated are presented and discussed in Section S4. Linear combination fits to the sample spectra presented in Figure 2 were performed using the reference spectra from Mg(B_3_H_8_)_2_, MgH_2_, and Mg(BH_4_)_2_ and are gathered in Table S2. The converged fits supply the contribution of each reference spectrum to the total signal and provide a good measure of the relative abundance. Analysis of the TFY signal shows that the bulk of the starting material is composed of ∼22% of Mg(B_3_H_8_)_2_ and ∼78% of MgH_2_, thereby confirming the expected Mg(B_3_H_8_)_2_-to-MgH_2_ ratio of 1:4. Furthermore, the bulk contribution of Mg(BH_4_)_2_ in the starting material is close to zero, indicating that the BH_4_^–^ species measured in ^11^B solid-state MAS NMR are likely to be attributed to NaBH_4_ rather than Mg(BH_4_)_2_. While NaB_3_H_8_ was used as the starting material for the synthesis of unsolvated Mg(B_3_H_8_)_2_ making this a plausible hypothesis, this observation requires further future studies. Due to the possibility of co-existing NaBH_4_ and Mg(BH_4_)_2_, we will discuss the conversion of Mg(B_3_H_8_)_2_ to BH_4_^–^ containing species without distinguishing the separate contributions of NaBH_4_ from Mg(BH_4_)_2_ after the conversion. Additionally, Figure 2a clearly shows a subtle surface contribution of metallic Mg(0) in the form of a small, broad peak ∼1300 eV, well segregated from the edge step of MgH_2_ at ∼1302 eV. This is the first experimental report of a metallic contribution in a ball-milled M(B_3_H_8_)*_n_*, *n* = 1 and 2 compounds. We hypothesize that the friction-induced heat provided during the ball-milling process is sufficient to partially reduce the Mg-species. Because the Mg(B_3_H_8_)_2_ baseline material, which was also ball-milled, does not show any Mg(0) contribution, we infer that it is either the MgH_2_ that is being reduced or the high thermal conductivity of MgH_2_ successfully transferring the heat to the Mg(B_3_H_8_)_2_ leading to its partial decomposition. It is known that metallic Mg and H_2_ are in equilibrium with MgH_2_, with a H_2_ pressure of ∼1.0 × 10^–4^ bar at 127 °C (see thermodynamic data from NIST in Table S6).^62−64^ These results are consistent with the hypothesis that surface MgH_2_ is converted into Mg(0) during the ball milling process.

### Thermal Conversion of Unsolvated Mg(B_3_H_8_)_2_ to BH_4_-Species

**Figure 3:**
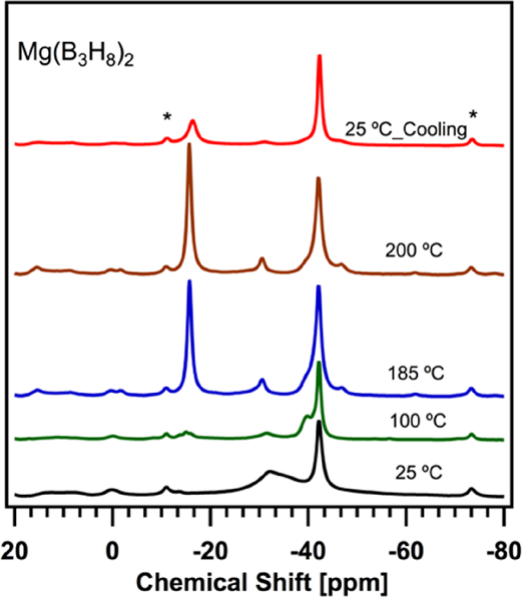
In situ variable-temperature ^11^B solid-state MAS NMR spectra of unsolvated Mg(B_3_H_8_)_2_ at 11.7 T, 5 kHz MAS at 25 (black spectrum), 100 (green spectrum), 185 (blue spectrum), 200 (brown spectrum), and 25 °C after cooling (red spectrum). The asterisks indicate the spinning side bands at −11.0 and −73.0 ppm.

**Figure 4:**
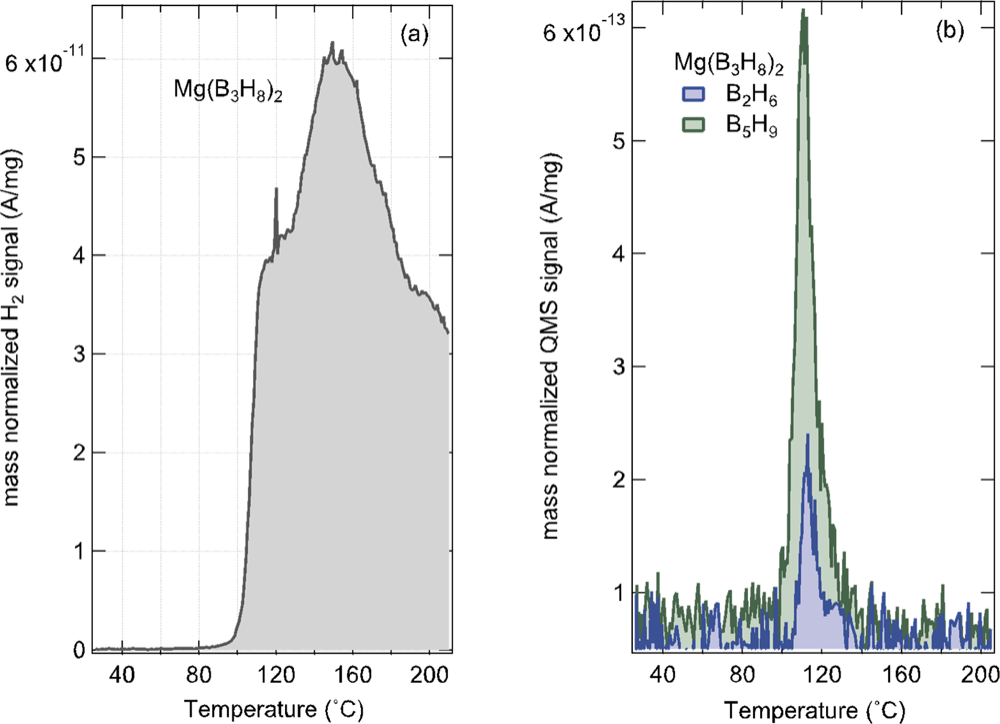
Mass normalized QMS signal recorded during the heating of unsolvated Mg(B_3_H_8_)_2_ with a heating ramp rate of 10 °C/min where panel (a) shows the thermal evolution of *m*/*z* = 2 (H_2_) and (b) the signal of *m*/*z* = 26 (B_2_H_4_) as the dominant fragment of diborane (B_2_H_6_) and *m*/*z* = 59 (B_5_H_4_) as the dominant fragment of pentaborane (B_5_H_9_).

We first investigated the thermal conversion of Mg(B_3_H_8_)_2_ by heating it from 25 to 200 °C recording the ^11^B solid-state NMR spectrum at 100, 185, and 200 °C, shown in Figure 3. Contributions from spinning side bands were identified at −11.0 and −73.0 ppm. During heating, the chemical shifts related to B_3_H_8_^–^ start decreasing at temperatures as low as 100 °C, while those related to BH_4_^–^, B_10_H_10_^2–^, B_12_H_12_^2–^, and borates increase up to 185 °C. The peak positions and intensities barely change from 185 to 200 °C suggesting that the thermal decomposition of the material is already complete at 185 °C. The temperature programmed desorption (TPD) of the sample in Figure 4a shows the release of H_2_ starting at ∼100 °C, confirming that the onset of the decomposition process occurs at ∼100 °C.

After heating the sample to 200 °C, it was rapidly quenched at 25 °C to collect the NMR chemical shift, for which the assignments of the species and the quantification are reported in Table 2. The thermal conversion of unsolvated Mg(B_3_H_8_)_2_ yields a mixture of BH_4_^–^, B_12_H_12_^2–^, and B_10_H_10_^2–^ in which BH_4_^–^ and B_10_H_10_^2–^ are the major species with net amounts (i.e., after subtraction of the BH_4_^–^ wt % from the starting material) of 22 and 32 wt %, respectively. The same solid products, BH_4_^–^, B_10_H_10_^2–^, and B_12_H_12_^2–^, were reported from the thermal decomposition of unsolvated KB_3_H_8_ studied by qualitative ^11^B solid-state MAS NMR.^15,17^ Interestingly, the thermal decomposition of solvated Mg(B_3_H_8_)_2_ with THF or diglyme leads to the formation of BH_4_^–^ and B_12_H_12_,^2^ but not B_10_H_10_^2–^.^22,65^ Therefore, solvents acting as Lewis bases, such as THF and diglyme, may affect the decomposition pathway toward the formation of B_12_H_12_^2–^, a thermodynamic sink, rather than B_10_H_10_^2–^.

**Table 2:** Chemical Shift Assignments and Quantification of the Chemical Composition after the Heat Treatment of Unsolvated mg(B_3_H_8_)_2_ Obtained from the Red Spectrum Shown in Figure 4^a^

^11^B chemical shift [ppm]	assignment	net wt %
–42.3, −42.0, −39.5, −46.0	BH_4_^–^	22
–30.8	B_3_H_8_^–^	3
–30.5, −0.10	B_10_H_10_^2–^	32
–16.4, −16.1	B_12_H_12_^2–^	16
17.0	BO*_x_*	15

a The BH_4_^–^ wt % reported in this table is the net wt %, i.e., the BH_4_^–^ wt % from the starting material is subtracted.

From TPD-MS, heating Mg(B_3_H_8_)_2_ not only released H_2_ but also diborane (B_2_H_6_) and pentaborane (B_5_H_9_) in the temperature range of 95–140 °C, as shown in Figure 4b. The *m*/*z* = 26 (B_2_H_4_) and *m*/*z* = 59 (B_5_H_4_) were monitored as representatives for B_2_H_6_ and B_5_H_9_, respectively. These species were identified by comparing the full fragmentation pattern with that from the NIST webbook, as can be seen in Figure S8. From our previous characterization using differential scanning calorimetry coupled with thermal gravimetric analysis of this unsolvated Mg(B_3_H_8_)_2_, a mass loss of 29 wt % was observed in the temperature range of 80–140 °C.^12^ Since the theoretical hydrogen content of Mg(B_3_H_8_)_2_ was 15 wt %, we concluded the formation of additional gases, such as B_2_H_6_ and B_5_H_9_, together with H_2_.^12^ The release of B_2_H_6_ and B_5_H_9_ was also reported during the thermal decomposition of unsolvated KB_3_H_8_ and NaB_3_H_8_.^11,15,17^

In the case of solvated Mg(B_3_H_8_)_2_ with diglyme, however, only the formation of B_5_H_9_ was reported.^65^ Therefore, it can be inferred that the decomposition of unsolvated Mg(B_3_H_8_)_2_ starts with the rupture of the B–H–B bridging bonding giving rise to BH_4_^–^ and B_2_H_4_, which polymerizes into B_2_H_6_ and B_5_H_9_ to further form B_12_H_12_^2–^ and B_10_H_10_^2–^ by condensation reactions. While *m*/*z* values characteristic for B_2_H_4_ were specifically scanned, these *m*/*z* values overlap with those for B_2_H_6_ and B_5_H_9_, and B_2_H_4_ is very unstable and could have reacted with other decomposition intermediates on its way to the mass spectrometer.^66^ For these reasons, the presence of B_2_H_4_ cannot be confirmed nor excluded in this work.

The fact that the thermal decomposition of unsolvated Mg(B_3_H_8_)_2_ yields a low BH_4_^–^ conversion of ∼22 wt % and is accompanied with the loss of B in the form of B_12_H_12_, B_2_H_6_, and B_5_H_9_ limits the range of Mg(B_3_H_8_)_2_ applications. In the pursuit of maximizing the conversion to BH_4_^–^ while minimizing the mass loss of the sample, activated (through ball milling) MgH_2_ was mixed with the Mg(B_3_H_8_)_2_, and the following section will show that it is a viable strategy for the selective formation of BH_4_^–^.

### The Role of MgH_2_ in the Thermal Conversion of Mg(B_3_H_8_)_2_ to BH_4_^–^

#### Thermal Conversion in a Closed System

**Figure 5:**
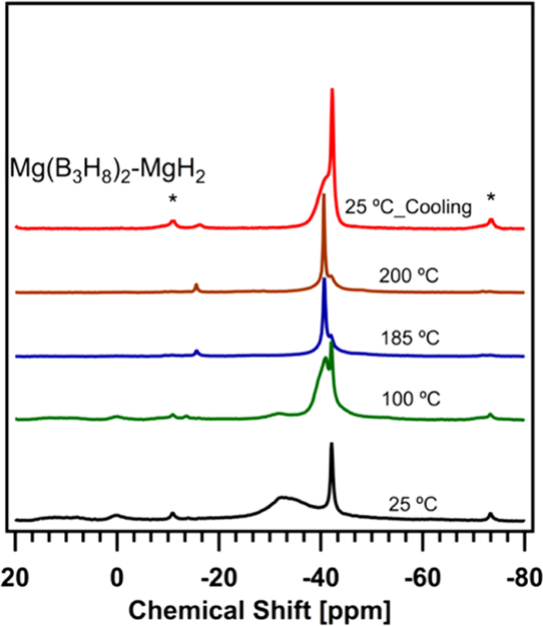
In situ variable-temperature ^11^B solid-state MAS NMR of Mg(B_3_H_8_)_2_-MgH_2_ at 11.7 T, 5 kHz MAS at 25 (black spectrum), 100 (green spectrum), 185 (blue spectrum), 200 (brown spectrum), and 25 °C after cooling (red spectrum). The asterisks indicate the spinning side bands at −11.0 and −73.0 ppm.

Following the same measurement procedure as the as-synthesized Mg(B_3_H_8_)_2_, in situ variable-temperature ^11^B solid-state MAS NMR spectra of the Mg(B_3_H_8_)_2_-MgH_2_ system were recorded at 100, 185, and 200 °C as shown in Figure 5. Like the patterns shown in Figure 4, the signal of B_3_H_8_^–^ starkly decreases already at 100 °C while the BH_4_^–^-related peaks increase up to 185 °C, after which no significant change occurs suggesting that the conversion is complete at that temperature. Unlike the conversion of unsolvated Mg(B_3_H_8_)_2_, the peaks related to B_10_H_10_^2–^, B_12_H_12_^2–^, and borates do not visibly arise during the heating of the MgH_2_-containing material. After cooling the heated sample, the NMR spectrum is collected (upper red spectrum in Figure 5), and the peaks are assigned to species that are quantified, as summarized in Table 3. The net conversion of BH_4_^–^ is ∼88 wt %. Because the 22 wt % of BH_4_^–^ from the starting material is subtracted to obtain 88 wt % and there is a clear absence of the B_3_H_8_^–^ signal from Figure 5, we conclude that this 88 wt % is equivalent to a complete conversion to BH_4_^–^ in this case. Not only is this number 4 times as much as in the absence of MgH_2_, but it is also the highest conversion to BH_4_^–^ occurring at ≤200 °C in this study. Indeed, the wt % of B_12_H_12_^2–^ was decreased from 16 wt % without MgH_2_ to 2 wt % in the presence of MgH_2_. Therefore, the presence of MgH_2_ selectively leads to BH_4_^–^ as the main decomposition product of the thermal decomposition of Mg(B_3_H_8_)_2_. To provide additional insight into the conversion of BH_4_^–^ as a function of temperature, we quantified the species at 100 °C and the values are reported in Table S5. The net formation of BH_4_^–^ (i.e., after subtraction of the BH_4_^–^ wt % from the starting material) is ∼59 wt %, indicating that BH_4_^–^ is the major product at a temperature as low as 100 °C and within 1 h (from the ramping rate of 10 °C/min used in the NMR experiments).

**Table 3:** Chemical Composition after the Heat Treatment of Mg(B_3_H_8_)_2_-MgH_2_ Obtained from the Chemical Shifts Shown in Red in Figure 5^a^

^11^B chemical shift [ppm]	assignment	net wt %
–42.2, −40.6	BH_4_^–^	88
–16.0	B_12_H_12_^2–^	2

a The BH_4_^–^ wt % reported in this table is the net wt %, i.e., the BH_4_^–^ wt % from the starting material is subtracted.

Interestingly, the presence of borates species (BO*_x_*) was not observed after heating Mg(B_3_H_8_)_2_-MgH_2_ (see Table 3) while a content of 15 wt % was found for the heat treatment of Mg(B_3_H_8_)_2_ as shown in Table 2. While 15 wt % is higher than expected (probably due to accidental exposure to some air/moisture), the “absence” of BO*_x_* after heating of Mg(B_3_H_8_)_2_-MgH_2_ suggests that the BO*_x_* is reduced by MgH_2_ following the reaction Mg(B_3_H_8_)_2_ + MgH_2_ + BO*_x_* → Mg(BH_4_)_2_ + Mg(OH)_2_. The fact that B is not “lost” through the formation of BO*_x_* when the Mg(B_3_H_8_)_2_-MgH_2_ system is heated increases the overall BH_4_^–^ yield. The reduction of BO*_x_* with MgH_2_ has been recently demonstrated at room temperature and even exploited in the synthesis of NaBH_4_ from NaBO*_x_* ball-milled with MgH_2_.^67−70^

For the first time, the combination of this high conversion percentage occurring at ≤200 °C, within 1 h, and without the external supply of H_2_ is an unprecedented performance for M(B_3_H_8_)*_n_*-MH*_n_*, *n* = 1 and 2 systems. Indeed, the KB_3_H_8_-KH system was converted to 36 mol % of BH_4_^–^ at 150 °C under 382 bar H_2_ after 24 h, while the Mg(B_3_H_8_)_2_·THF-MgH_2_ was reported to have a conversion to 9 mol % BH_4_^–^ at 200 °C under 50 bar of H_2_ after 2 h. Therefore, the unsolvated Mg(B_3_H_8_)_2_-MgH_2_ system boosted (i) the conversion to BH_4_^–^ without the use of external hydrogen pressure and (ii) the time of conversion.

**Figure 6:**
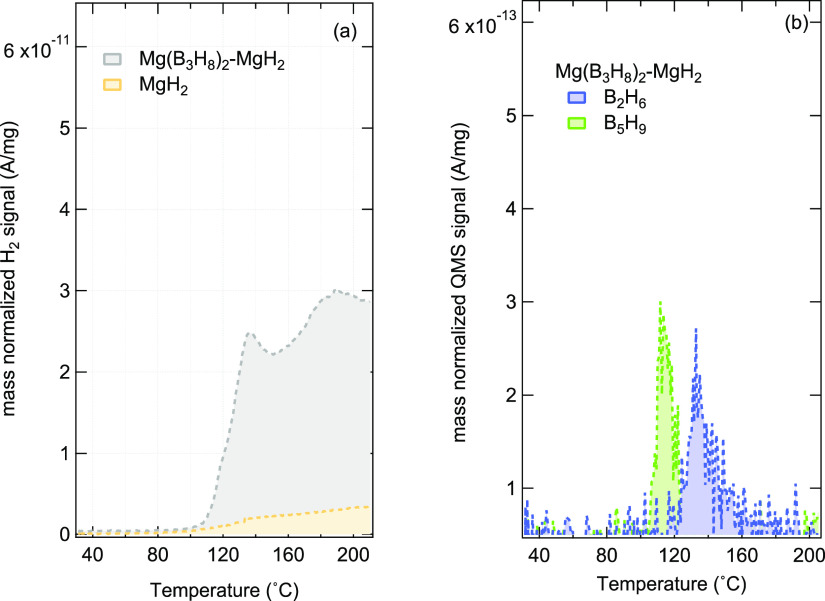
Mass normalized QMS signal recorded during the heating of Mg(B_3_H_8_)_2_-MgH_2_ with a heating ramp rate of 10 °C/min where panel (a) shows the thermal evolution of *m*/*z* = 2 (H_2_) and panel (b) shows the signal of *m*/*z* = 26 (B_2_H_4_) as the dominant fragment of diborane (B_2_H_6_) and *m*/*z* = 59 (B_5_H_4_) as the dominant fragment of pentaborane (B_5_H_9_). Note that the scale is the graph that is kept the same as Figure 4 for a clear comparison.

From the TPD-MS experiments performed on Mg(B_3_H_8_)_2_-MgH_2_, shown in Figure 6, the decomposition starts at ∼110 °C, which is similar to that of Mg(B_3_H_8_)_2_. The H_2_ signal recorded in Figure 6a confirms that in the temperature range considered, the dominant portion of H_2_ originates from Mg(B_3_H_8_)_2_ rather than MgH_2_. The H_2_ quantification from the areal integration of the signal was performed for Mg(B_3_H_8_)_2_ and Mg(B_3_H_8_)_2_-MgH_2_ and is presented in Table 4. Compared to the Mg(B_3_H_8_)_2_ sample, the amount of H_2_ released in the presence of MgH_2_ is almost halved. While the quantification of B_2_H_6_ and B_5_H_9_ was not performed, their associated *m*/*z* signal is significantly attenuated in the Mg(B_3_H_8_)_2_-MgH_2_ system, as shown in Figure 6b_._ It is further noteworthy and puzzling to see the release of B_5_H_9_ at lower temperatures than the release of B_2_H_6_, as it is widely accepted that B_5_H_9_ is formed from B_2_H_6_.^71−73^

**Table 4:** Gravimetric Capacity of H_2_ in wt % Extracted from the TPD-MS Data^a^

sample	normalized to the sample mass used (H_2_ (wt %))	normalized to the mass of Mg(B_3_H_8_)_2_ (H_2_ (wt %))
Mg(B_3_H_8_)_2_	0.31 ± 0.03	0.92 ± 0.09
Mg(B_3_H_8_)_2_-MgH_2_	0.14 ± 0.01	0.55 ± 0.06

a The signal was normalized to the mass of the sample used and normalized to the mass of the “active” sample (i.e., H_2_-containing species) using the molecular weights of Mg(B_3_H_8_)_2_, MgH_2_, and NaBr used in the synthesis, i.e., normalized to the molar mass of the active sample (see Table S3 for more details). Values apply for the desorption temperatures within 25 and 200 °C. The values are accurate within a 10% error.

Moreover, the concomitant presence of B_2_H_6_ and B_5_H_9_ was also observed by in situ variable-temperature ^11^B MAS NMR of Mg(B_3_H_8_)_2_ at 100 °C (red spectrum in Figure 3), and their quantification is provided in Table S3. Therefore, it is plausible that the conversion of B_2_H_6_ into B_5_H_9_ occurs at a time scale faster than seconds as the refreshing time of the MS and of the ^11^B MAS NMR each takes seconds and these techniques are not able to differentiate from B_2_H_6_ formation and its conversion process. This observation indicates that MgH_2_ not only promotes the selective B_3_H_8_-to-BH_4_^–^ conversion but also potentially affects the decomposition mechanism, either by trapping/inhibiting reaction intermediates or by favoring certain reaction steps at the expense of others. Indeed, the reaction of metal hydride with B_2_H_6_ has been exploited as a synthesis route for borohydride compounds.^27,38−41,74^ The Mg(B_3_H_8_)_2_-MgH_2_ system presented herein is the first example among M(B_3_H_8_)*_n_*, *n* = 1 and 2 compounds to give close to a full conversion to BH_4_^–^ at 200 °C in less than 1 h without the use of hydrogen pressure. Grinderlsev et al. have recently reported the partial hydrogenation of KB_3_H_8_-KH at 150 °C under 380 bar after 24 h yielding a mixture of BH_4_^–^, B_12_H_12_^2–^, B_10_H_10_^2–^, and B_9_H_9_^2–^.^11^ It appears that MgH_2_ is a better inhibitor than KH. This might be explained by the different reactivities of metal hydrides. MgH_2_ is a better nucleophilic and reactive agent than KH. The smaller size of Mg^2+^ than K^+^ leads the H^–^ anion to be more reactive toward B_3_H_8_^–^ so that the formation of BH_4_^–^ is not only faster but also more efficient. The high conversion percentage obtained by mixing MgH_2_ to Mg(B_3_H_8_)_2_ infers that MgH_2_ acts as the hydrogen donor to B–H–B bridge bonding during the decomposition of Mg(B_3_H_8_)_2_. Moreover, one reason for such high conversion to BH_4_^–^ upon heating Mg(B_3_H_8_)_2_-MgH_2_ may be explained by the activation of MgH_2_ with ball milling before mixing it with unsolvated Mg(B_3_H_8_)_2_.^41,75−78^ The ball milling process potentially decreases the particle size of MgH_2_ leading to an increase of the surface area and therefore a higher reactivity of MgH_2_ toward B_3_H_8_^–^. To confirm this hypothesis, the Mg(B_3_H_8_)_2_-MgH_2_ was pressurized and the conversion percentage and conditions were compared with the experiment without additional H_2_ pressure.

#### Thermal Conversion against 6 bar H_2_ Backpressure

**Figure 7:**
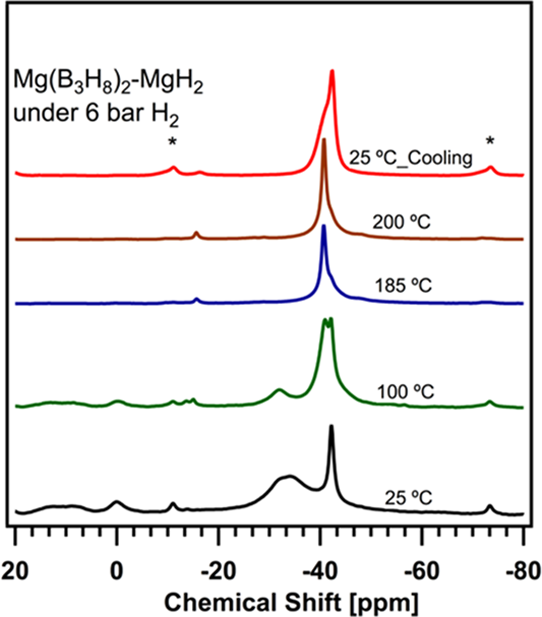
In situ variable-temperature ^11^B solid-state MAS NMR of Mg(B_3_H_8_)_2_-MgH_2_ under 6 bar H_2_ at 11.7 T, 5 kHz MAS at 25 (black spectrum), 100 (green spectrum), 185 (blue spectrum), 200 (brown spectrum), and 25 °C after cooling (red spectrum). The asterisks indicate the locations of spinning side bands at −11.0 and −73.0 ppm.

The Mg(B_3_H_8_)_2_-MgH_2_ system was hydrogenated under 6 bar of H_2_ at 25 °C in a closed NMR ceramic rotor, and Figure 7 shows the ^11^B solid-state MAS NMR spectrum collected at 25 °C (black line) and after heating the sample using the same procedure used for the two previous samples. The chemical shifts collected at 100, 185, and 200 °C as well as after cooling the sample to 25 °C after heating are also shown in Figure 7. The quantification of the species of the starting material is reported in Table S4. The quantification from the integrated intensity from the sample cooled after heating is reported in Table 5. A B_3_H_8_^–^-to-BH_4_^–^ conversion of ∼85 wt % was achieved during the hydrogenation of Mg(B_3_H_8_)_2_ at 200 °C. Because this conversion percentage is very similar to that without H_2_ pressure, we conclude that the presence of MgH_2_ plays the main role in the full conversion of B_3_H_8_^–^ to BH_4_^–^. Therefore, in the case of unsolvated Mg(B_3_H_8_)_2_, it is possible to obtain BH_4_^–^ under moderate conditions of temperature and hydrogen pressure.

**Table 5:** Chemical Composition after the Heat Treatment of Mg(B_3_H_8_)_2_-MgH_2_ under 6 bar of H_2_ Obtained from the Red Chemical Shift Shown in Figure 7^a^

^11^B chemical shift [ppm]	assignment	net wt %
–42.3, −40.6, −48.1	BH_4_^–^	85
–29.8, −27.0, −0.20	B_3_H_8_^–^, B_10_H_10_^2–^	less 1%
–16.0	B_12_H_12_^2–^	2
17.0	BO*_x_*	2

a The BH_4_^–^ wt % reported in this table is the net wt %, i.e., the BH_4_^–^ wt % from the starting material is subtracted.

### Mechanistic Considerations

Based on the NMR, TPD, and XAS measurement, the intermediates formed without MgH_2_ are H_2_, B_10_H_10_^2–^, B_12_H_12_^2–^, B_2_H_6_, and B_5_H_9_, and with MgH_2_, only negligible amounts of H_2_, B_2_H_6_, and B_5_H_9_ were detected. For the Mg(B_3_H_8_)_2_ decomposition, there are too many possibilities for reactions to yield the said reaction products. Furthermore, the role of MgH_2_ is the focus of this study; hence, we propose a combination of the following reactions to occur in the hydrogenation of Mg(B_3_H_8_)_2_ in the presence of MgH_2_.
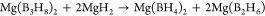


In this case, this back conversion to BH_4_ is not complete. Therefore, an excess of MgH_2_ is necessary to provide the hydrogen atoms necessary to form BH_4_^–^:
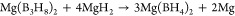


This implies here that Mg^2+^ has been reduced to Mg^0^. While XAS has not confirmed the presence of Mg^0^ after heating Mg(B_3_H_8_)_2_ with MgH_2_, additional analysis needs to be performed to be conclusive. A possible “trapping” mechanism for the volatile products diborane and pentaborane is
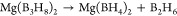

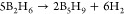

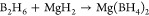


Pentaborane can in principle also be trapped by MgH_2_ to form first a reactive species such as Mg(B_5_H_10_)_2_, which can then react rapidly further to form the closoborate ions B_10_H_10_^2–^ and B_12_H_12_^2–^. Theoretical studies of possible reaction pathways to form closoborates are under way.

## Conclusions

In this work, we investigated the thermal decomposition of solvent-free Mg(B_3_H_8_)_2_ and its conversion into decompsotion products. It was observed that these pristine samples started to decompose at ∼100 °C. Heating the unsolvated Mg(B_3_H_8_)_2_ to 200 °C results in a low net conversion to BH_4_^–^ of 22 wt %, releasing volatile B_2_H_6_ and B_5_H_9_ and forming very stable solid closoboranes. The mass loss of these borane species is unfeasible for applications requiring cyclability.

The addition of activated MgH_2_ was added to the solvent-free Mg(B_3_H_8_)_2_, which led to a complete conversion to BH_4_^–^ occurring at ∼100 °C within 1 h, which is the first system to be reported of this achievement. This work demonstrates that the addition of activated MgH_2_ leads to the selective conversion of BH_4_^–^, with only traces of closoboranes formed. The result is of significant importance as it is the first to be reported in the literature and expands the use of M(B_3_H_8_)*_n_*-MH*_n_*, *n* = 1 and 2 to a series of applications. This work has shown that MgH_2_ acts (i) as an inhibitor for the volatile B_2_H_6_ and B_5_H_9_, thereby maximizing the mass retention of the Mg(B_3_H_8_)_2_-MgH_2_ system, and (ii) as the hydrogen donor for the B_3_H_8_-to-BH_4_^–^ conversion to occur. This was confirmed when the thermal conversion of Mg(B_3_H_8_)_2_-MgH_2_ remained ∼85 wt % when exposed to 6 bar of H_2_, compared to ∼88 wt % without excess H_2_ available. The exact role of MgH_2_ in the low-temperature B_3_H_8_-to-BH_4_^–^ conversion process, or more generally its role as a H_2_ donor, will be scrutinized in future studies, especially in terms of MgH_2_ activation, which seems to play a paramount part in achieving the high conversion percentage.

Overall, these findings can increase the cyclability of B_3_H_8_^–^-based electrolytes for batteries, liquid hydrogen carriers, and H_2_ long-term storage. Furthermore, by inhibiting toxic volatile by-products, such as B_2_H_6_ and B_5_H_9_, the addition of MgH_2_ provides a green chemistry approach to using B_3_H_8_^–^-containing precursors for vapor-phase deposition techniques as well as improving the purity of metal boride films deposited.

## Supporting Information Available

Synthesis of the precursors for the preparation of unsolvated Mg(B_3_H_8_)_2_; materials; synthesis of TBABH_4_; synthesis of TBAB_3_H_8_; synthesis of NaB_3_H_8_; ceramic rotor for in situ variable temperature and hydrogenation ^11^B solid-state MAS NMR experiments; ^1^H solid-state MAS NMR experiments of as-synthesized Mg(B_3_H_8_)_2_-MgH_2_ at 25 °C; ^11^B solid-state MAS NMR characterization of as-synthesized NaB_3_H_8_; solution ^11^B NMR of as-synthesized NaB_3_H_8_; ^11^B solid-state magic angle spinning nuclear magnetic resonance spectroscopy (^11^B solid-state MAS NMR); characterization of Mg(B_3_H_8_)-MgH_2_ batch 2 and comparison with batch 1; X-ray diffraction (XRD) characterization of batch 2 of Mg(B_3_H_8_)_2_-MgH_2_; solution ^11^B NMR of Mg(B_3_H_8_)-MgH_2_ for the XANES experiment; TPD-MS, TGA, and DSC characterization of batch 2; quantification extracted from the XANES experiment of Mg(B_3_H_8_)_2_-MgH_2_; molar masses used for the mass normalization of the TPD data; quantification obtained by ^11^B solid-state MAS NMR of Mg(B_3_H_8_)_2_-MgH_2_ under 6 bar of H_2_ at 25 °C before in situ variable temperature ^11^B solid-state MAS NMR experiments; quantification obtained from ^11^B solid-state MAS NMR of Mg(B_3_H_8_)_2_-MgH_2_ at 100 °C; and hydrogen pressure obtained for the decomposition of MgH_2_ (PDF)
