# Supplementary material for: Thermal Conversion of Unsolvated Mg(B3H8)2 to BH4– in the Presence of MgH2
Source: ACS Appl Energy Mater. 2021 Apr 2;4(4):3737–47. doi: 10.1021/acsaem.1c00159 (PMC10156084; doi:10.1021/acsaem.1c00159)
Supplement: Supplementary file 1 — ae1c00159_si_001.pdf [file ae1c00159_si_001.pdf]

Supporting Information

Thermal conversion of unsolvated $Mg(B_3H_8)_2$ to BH_4^- in the presence of MgH_2

Angelina Gigante[1], Noemi Leick[2*], Andrew Lipton[3], Ba Tran[4], Nicholas A. Strange[2,5], Mark Bowden[3], Madison B. Martinez[2], Romain Moury[1,6], Thomas Gennett[2,7], Hans Hagemann[1], Tom S. Autrey[3]

[1]*Département de Chimie Physique, Université de Genève, 30, quai E. Ansermet, 1211 Geneva 4, Switzerland.*
[2]*National Renewable Energy Laboratory, 15013 Denver W Pkway, Golden, CO 80401, USA.*
[3]*Environmental Molecular Division, Earth and Biological Sciences Directorate, USA Pacific Northwest National Laboratory, Richland, WA 99354, USA.*
[4]*Physical Sciences Division, Physical and Computational Sciences Directorate, Pacific Northwest National Laboratory, Richland, WA 99354, USA.*
[5]*SLAC National Accelerator Laboratory, 2575 Sand Hill Road, Menlo Park CA 94025, USA*
[6]*Institut des Molécules et des Matériaux du Mans, UMR 6283 CNRS, Le Mans Université, Avenue Olivier Messiaen, 72085 Le Mans Cedex 9, France.*
[7]*Chemistry Department, Colorado School of Mines, 1012 14th street, Golden CO, 80401 USA.*

*corresponding author(s): noemi.leick@nrel.gov

Table of Contents

S1 Synthesis of the precursors for the preparation of unsolvated $Mg(B_3H_8)_2$……2

S1.1Materials……2

S1.2 Synthesis of $TBABH_4$……2

S1.3 Synthesis of $TBAB_3H_8$……2

S1.4 Synthesis of NaB_3H_8……3

S2. Ceramic rotor for in situ variable temperature and hydrogenation ^{11}B solid-state MAS NMR experiments……3

S2.1. ^1H solid state MAS NMR experiments of as synthesized $Mg(B_3H_8)_2$-MgH_2 at 25°C……4

S3 ^{11}B solid state MAS NMR characterization of as synthesized NaB_3H_8……4

S3.1. Solution ^{11}B NMR of as synthesized NaB_3H_8……4

S3.2 ^{11}B solid-state magic angle spinning nuclear magnetic resonance spectroscopy (^{11}B solid-state MAS NMR)……5

S4 Characterization of $Mg(B_3H_8)$-MgH_2 batch 2 and comparison with batch1……5

S4.1. X-ray diffraction (XRD) characterization of batch 2 of $Mg(B_3H_8)_2$-MgH_2……5

S4.2 Solution ^{11}B NMR of $Mg(B_3H_8)$-MgH_2 for XANES experiment……6

S4.3 TPD-MS, TGA and DSC characterization of batch 2……7

S5. Quantification extracted from the XANES experiment of $Mg(B_3H_8)_2$-MgH_2……8

S6. Molar masses used for the mass normalization of the TPD data……9

S7. Quantification obtained by ^{11}B Solid-state MAS NMR of $Mg(B_3H_8)_2$-MgH_2 under 6 bar of H_2 at 25°C before in situ variable temperature ^{11}B solid state MAS NMR experiments……9

S8. Quantification obtained from ^{11}B Solid-state MAS NMR of $Mg(B_3H_8)_2$-MgH_2 at 100°C……9

S9. Hydrogen pressure obtained for the decomposition of MgH_2……10

S10. References……10

S1 Synthesis of the precursors for the preparation of unsolvated $Mg(B_3H_8)_2$

Two batches of unsolvated $Mg(B_3H_8)_2$ were prepared for the investigation of its thermal conversion. We will call batch 1 to indicate the unsolvated $Mg(B_3H_8)_2$ used for the ^{11}B solid-state MAS NMR and TPD-MS experiments, and batch 2 to prepare unsolvated $Mg(B_3H_8)_2$ for XANES analysis. Both batches were prepared by the following protocol. The cleaning protocol used for the jars and balls after the ball milling of the activation of MgH_2, the synthesis of unsolvated $Mg(B_3H_8)_2$ and $Mg(B_3H_8)_2$-MgH_2 was the following: a few mg of Silica gel (SiO_2) from Sigma Aldrich (Technical grade, pore size 60 Å, 70-230 mesh, 63-200µm) were introduced in the jars together with distilled water. The mixture was milled for 2h at 450rpm with a milling period of 5 min and 1min break. The procedure was repeated until the solid residue from the syntheses was removed. Then, the jars were cleaned by distilled water, scrub with soap, and rinse with acetone. Finally, the jars were dried in oven overnight.

S1.1 Materials

Tetra-n-butylammonium chloride salt, $(C_4H_9)_4NCl$ (TBACl) (SIGMA ALDRICH, purity ≥ 97%), sodium borohydride $NaBH_4$ (ACROS, purity 99%), sodium tetraphenylborate salt, $(C_6H_5)_4BNa$ ($NaBPh_4$, purity ≥ 99.5%) (FLUKA), isopropanol (Fisher Chemical, analytical reagent grade), Magnesium hydride, MgH_2 (ALFA Aeser, 98% purity) and magnesium bromide, $MgBr_2$ (SIGMA ALDRICH, 98% purity), were used without any previous treatment. Dry Dichloromethane (DCM) was passed through activated 3 Å molecular sieves over a column of activated silica and it was provided by the department of Organic Chemistry of the University of Geneva, Switzerland. After the drying process of DCM, the content of H_2O was found to be 2.0 ppm. All compounds were stored and handled in an Argon filled glove box (H_2O < 0.1 ppm, O_2 < 0.1 ppm), and all solvents were used under nitrogen flow. Ball milling (FRITSCH Pulverisette 7 Premium Line) was used for tetrabutylammonium borohydride, $(C_4H_9)_4NBH_4$ ($TBABH_4$). Autoclaves for solvothermal synthesis of $TBAB_3H_8$ were home-built and involved a heating oven from BINDER GmbH using the R3 controller. The device can be operated in a temperature range of 5°C to 300°C. PM 100 CM (Retsch/grandprix 2008) to prepare unsolvated $Mg(B_3H_8)_2$, $Mg(B_3H_8)_2$-MgH_2 and to activate MgH_2. Nitrogen-vacuum line with standard Schlenk flasks were used for the synthesis of NaB_3H_8 according to the procedure developed by Moury et al.[1-2]

S1.2 Synthesis of $TBABH_4$

$(C_4H_9)_4NBH_4$ ($TBABH_4$) was prepared by milling 3 eq. (0.1450 g) of $NaBH_4$ with 1 eq. (0.3550 g) of TBACl in a 20mL stainless steel air tight milling vessels with five stainless steel balls with 10 mm diameters (balls-to-reactants mass ratio ~40) for batch 1 and 65mL stainless steel air tight milling vessels with 3 stainless steel balls with 10 mm diameters (balls-to-reactants mass ratio ~40) for batch 2. The milling was done at room temperature during 60 min at 500 rpm (15 min milling/5 min break). The jars were filled inside the glovebox. The white powder was then stored inside the Argon filled glovebox.

S1.3 Synthesis of $TBAB_3H_8$

$(C_4H_9)_4NB_3H_8$ ($TBAB_3H_8$) was prepared according to the following procedure: The mixture of $TBABH_4$-2eq. $NaBH_4$-1eq.NaCl (0.40 g) was placed in a ~14 mL Teflon-lined autoclave with 7 ml of dry DCM and tightened inside the glove box. The autoclave was placed in the oven at 80°C for 16 h. After the reaction, the reactor was opened outside of the glovebox and the white solid residues (NaCl and $NaBH_4$) were filtered. The solid was washed 3 times with 3 ml of dry DCM. Then, the liquid was evaporated under dynamic vacuum at 45°C. The resulting white solid was washed several times with 3 ml of distilled water and dried under vacuum for several hours at room temperature. The synthesis can also be performed with a bigger autoclave: 10 g (30 mmol TBACl and 90 mmol $NaBH_4$) of the as-obtained mixture in the

first step is placed in the Teflon reactor with 110 mL of dry DCM. The autoclave was heated for 16 h at 80°C and opened outside of the glovebox at the end of reaction.

S1.4 Synthesis of NaB_3H_8

In a 250ml round-bottom Schlenk flask, 5.94 g of $TBAB_3H_8$ was placed and dissolved in 75 ml of isopropanol (solubilization was not completed). An excess of $TBAB_3H_8$ was utilized because it can be easily removed using dry DCM after the synthesis. 5.42 g of sodium tetraphenylborate ($NaBPh_4$) was used in a 100 ml Erlenmeyer flask and dissolved in 50 ml of isopropanol and mixed at room temperature until the salt was completely dissolved. The clear solution of sodium tetraphenylborate was slowly added to the $TBAB_3H_8$ solution under nitrogen flow. The reaction mixture was allowed to stir for several hours at room temperature. During the addition, a quick formation of a dense white precipitate (tetrabutylammonium tetraphenylborate, $TBABPh_4$) was observed. The filtration was done using fritte 4P linked to a 250ml round-bottom Schlenk flask, dried under vacuum prior to use. The reaction mixture was transferred in the fritte under nitrogen flow. After filtration, the solid residue was washed several times with 5 ml of isopropanol. The solvent was removed under vacuum at room temperature and the as-obtained white solid was washed several times with 5 ml of dry DCM until all $TBAB_3H_8$ was removed from the solid product. The solid was isolated from the solution and dried under vacuum at 50°C for several hours.

S2. Ceramic rotor for in situ variable temperature and hydrogenation ^{11}B solid-state MAS NMR experiments

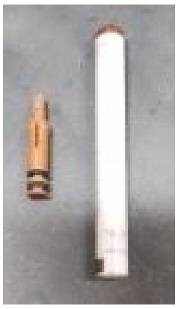

Figure S1. Picture of the ceramic rotor used for ^{11}B solid state MAS NMR experiments at EMSL user facility, Pacific Northwest National Laboratory (PNNL), USA.

S2.1. ^1H solid state MAS NMR experiments of as synthesized $Mg(B_3H_8)_2$-MgH_2 at 25°C

^1H solid state MAS NMR experiments were performed at 25°C before and after introducing 6bar of H_2 inside the ceramic rotor containing the as synthesized $Mg(B_3H_8)_2$-MgH_2 in order to detect the presence of H_2. The results are shown in **Figure S2**. The sharp peak at 4.3ppm (red spectrum in **Figure S2**) indicates the presence of H_2.

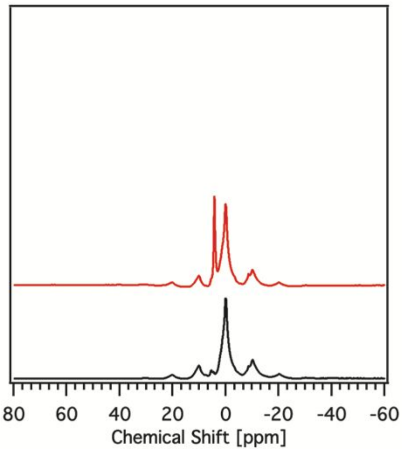

Figure S2. ^{1}H solid state MAS NMR spectra of $Mg(B_3H_8)_2$-MgH_2 before 6bar H_2 and after introducing 6 bar H_2 at 11.7T, 5kHz MAS and 25°C.

S3 ^{11}B solid state MAS NMR characterization of as synthesized NaB_3H_8
S3.1. Solution ^{11}B NMR of as synthesized NaB_3H_8

Solution ^{11}B NMR was performed for the as synthesized NaB_3H_8 used to make the batch 1. The sample was dissolved in CD_3OD and the result is shown in **Figure S3**. The ^{11}B NMR spectrum was acquired on Bruker ADVANCE III HD-NanoBay 300 MHz spectrometer. The chemical shifts of ^{11}B were referred to BF_3•OEt_2 (δ = 0 ppm) at 20 °C. ^{11}B was measured at 160.4 MHz. The samples were prepared in the glove box with H_2O < 0.1 ppm, O_2 < 0.1 ppm. Methanol-d_4 (CD_3OD) (D= 99.8%) was bought from Sigma Aldrich. The solvent was stored in the glovebox without any previous treatment. The experiment was performed with a 90° pulse width of 6 µs. The solution ^{11}B NMR spectrum was recorded with 64 scans by using a relaxation delay of 15s. The experiment was run at 20°C.
The peak observed at -30.6 ppm was assigned to $B_3H_8^-$ and is in agreement with the literature.[1-2] No traces of $NaBPh_4$ were observed, which indicates that the as synthesized NaB_3H_8 does not have $NaBPh_4$ as impurity. It is worth nothing that this batch was used to prepare unsolvated $Mg(B_3H_8)_2$, which was then investigated by ^{11}B solid-state magic angle spinning nuclear magnetic resonance spectroscopy (^{11}B solid-state MAS NMR), also known as batch 1.

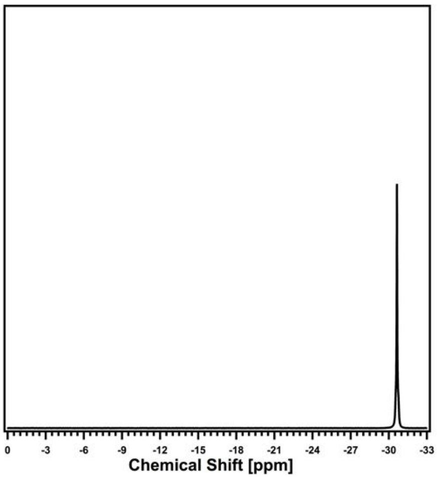

Figure S3. Solution ^{11}B NMR spectrum of as synthesized NaB_3H_8 at 20°C.

S3.2 ^{11}B solid-state magic angle spinning nuclear magnetic resonance spectroscopy (^{11}B solid-state MAS NMR)

^{11}B solid-state MAS NMR spectrum of as synthesized NaB_3H_8 was collected at 25°C to investigate the origin of BH_4^-, which was observed in the ^{11}B Solid-state MAS NMR spectrum of unsolvated $Mg(B_3H_8)_2$. The result is represented in **Figure S4** and the quantification of the species is reported in **Table S1**. The presence of $B_3H_8^-$ is revealed by the peaks at -34.3, -33.0, and -32.0 ppm with a content of 80%. The signal at -42.6ppm indicates the presence of BH_4^-, which is estimated to be 15%.

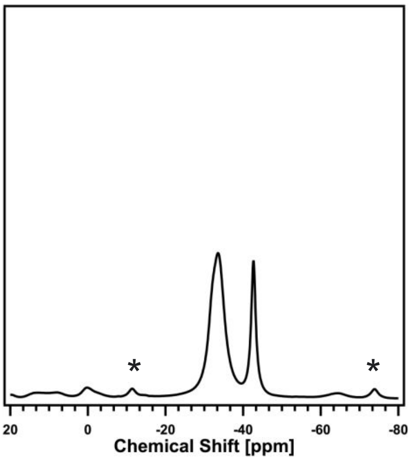

Figure S4. ^{11}B solid-state MAS NMR spectrum of as synthesized NaB_3H_8 at 11.7T, 5kHz MAS and 25°C. The asterisks * indicate the spinning side bands at -11.0 and -73.0 ppm.

^{11}B Chemical shift [ppm]	Assignment	wt%
-34.3, -33.0, -32.0	$B_3H_8^-$	80
-42.6	BH_4^-	15
-0.1, 8.0, 13.0	BO_x	5

Table S1. Chemical composition of the as synthesized NaB_3H_8 obtained by ^{11}B Solid-state MAS at 11.7T, 5kHz MAS and 25°C.

S4 Characterization of $Mg(B_3H_8)$-MgH_2 batch 2 and comparison with batch1
S4.1. X-ray diffraction (XRD) characterization of batch 2 of $Mg(B_3H_8)_2$-MgH_2

X-ray diffraction of batch 2 was compared with the that of batch 1. Powders for XRD were sealed under inert gas in thin-walled glass capillaries (Charles Supper Co., MA) and mounted in a Rigaku D/Max Rapid II diffractometer. X-rays from a Cr rotating anode source (λ = 2.2910 Å) were collimated through a 300 µm diameter aperture and the diffracted intensities recorded on a large 2D image plate. The 2D images were integrated using Rigaku software to give conventional 1D diffractograms for comparison with reference patterns from the International Centre for Diffraction Data. **Figure S5** shows a comparison between the as synthesized $Mg(B_3H_8)_2$-MgH_2 for ^{11}B solid-state MAS NMR and TPD-MS experiments (batch 1, red line) with as synthesized $Mg(B_3H_8)_2$-MgH_2 (batch 2, black line) for XANES experiment. The XRD experiment was performed at 25°C. Both samples show the concomitant presence of NaBr and

$MgBr_2$, which may indicate that the metathesis reaction was not completed. Some of the $MgBr_2$ peaks are broadened or shifted, and this is believed to arise from defects and stresses introduced by ball-milling.

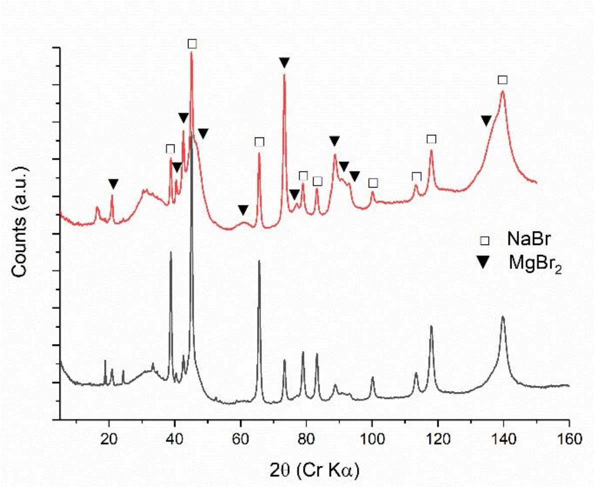

Figure S5. XRD pattern of $Mg(B_3H_8)_2$-MgH_2 for ^{11}B Solid-state MAS NMR and TPD-MS experiments (red line) and $Mg(B_3H_8)_2$-MgH_2 for XANES experiment (black line) at 25°C. The arrows ▼ indicate the $MgBr_2$ and the squares represent NaBr.

S4.2 Solution ^{11}B NMR of $Mg(B_3H_8)$-MgH_2 for XANES experiment

Solution ^{11}B NMR spectra were acquired on Varian Unity Innova 500 MHz spectrometer. The chemical shifts of ^{11}B were referred to BF_3•OEt_2 ($\delta = 0$ ppm) at 20 °C. ^{11}B was measured at 160.4 MHz. The sample was prepared in the glove box with $H_2O < 0.1$ ppm, $O_2 < 0.1$ ppm. Dimethyl sulfoxide-d_6 ($(CD_3)_2SO$) (D= 99.9%) was purchased from Cambridge Isotope Laboratories. The solvent was stored in the glovebox without any previous treatment.

The experiment was performed with a 90° pulse width of 6 µs. The ^{11}B NMR spectrum was recorded with 64 scans by using a relaxation delay of 15s at 20°C.

Figure S6 shows the solution ^{11}B NMR of as synthesized $Mg(B_3H_8)$-MgH_2 called batch 2 for XANES experiments. The NMR spectrum reveals the signal at -6.0 ppm, which indicates that $NaBPh_4$ remains as residue after the synthesis of NaB_3H_8, the latter is the precursor for the synthesis of unsolvated $Mg(B_3H_8)_2$.[3]

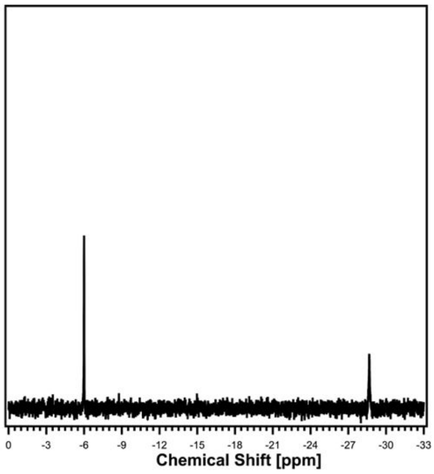

Figure S6. Solution ^{11}B NMR spectrum of as synthesized $Mg(B_3H_8)$-MgH_2 at 20°C.

S4.3 TPD-MS, TGA and DSC characterization of batch 2

Because solution ^{11}B NMR shows the presence of $NaBPh_4$ in batch 2, we performed TPD-MS of $NaBPh_4$ to assess its low-temperature thermal stability. The recorded fragmentation pattern at 150˚C is shown in **Figure S7** which is due to a partial decomposition of the compound. While it is possible that some of the decomposition products recorded in **Figure S9** react with the sample, it is noteworthy that the intensity of the pure material is low.

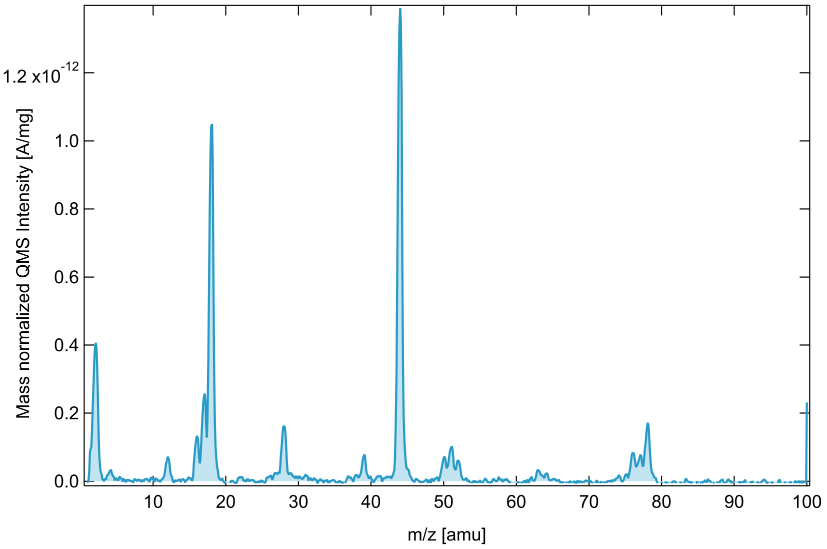

Figure S7. Fragmentation pattern of $NaBPh_4$ recorded at 150 ˚C using a quadrupole mass spectrometer (QMS) with the range of m/z=1-100.

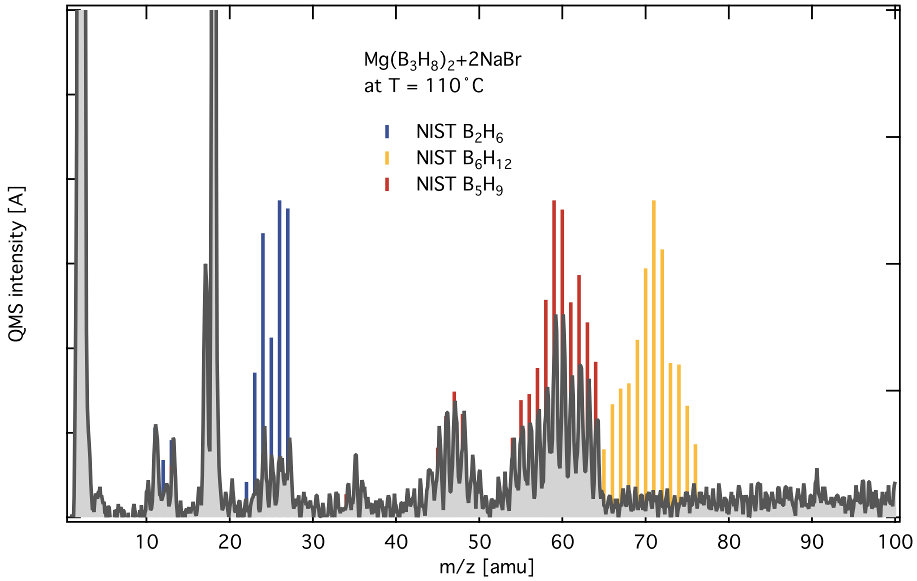

Figure S8. Fragmentation pattern of the gas-phase products detected at 110 ˚C using a quadrupole mass spectrometer (QMS) with the range of m/z=1-100. The bar charts are the fragmentation patterns of B_2H_6 (diborane), B_6H_{12} and B_5H_9 (pentaborane) as published by NIST. From this, we identified the presence of diborane and pentaborane in the gas-phase during heating of $Mg(B_3H_8)$.

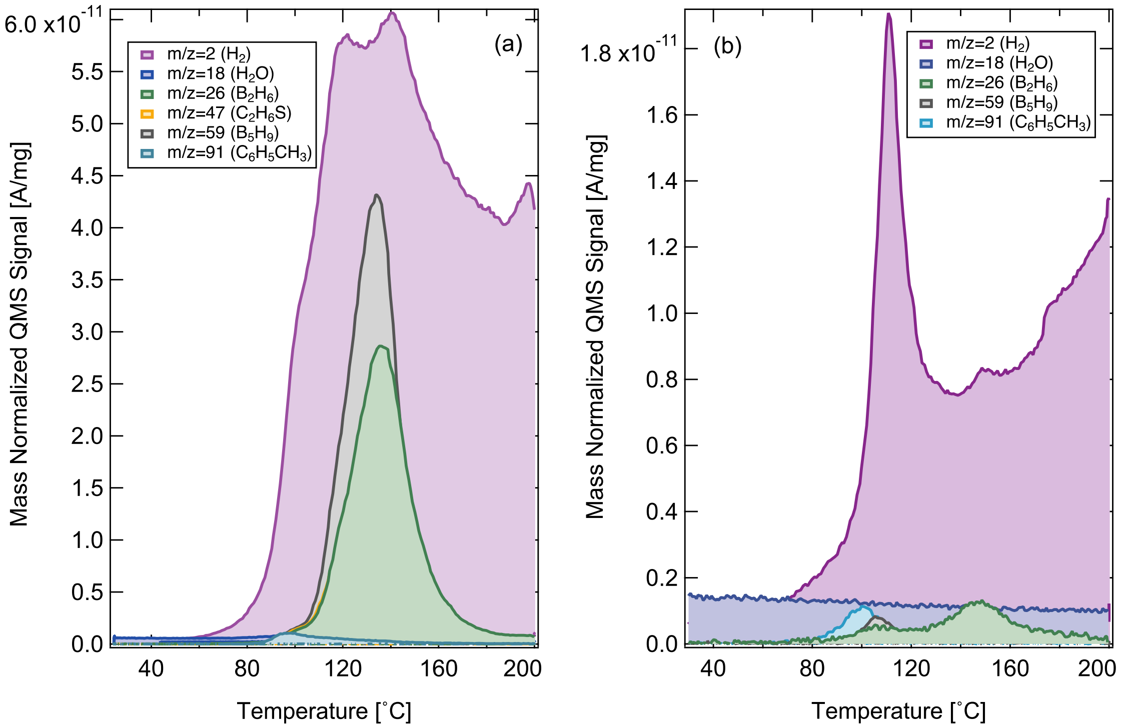

Figure S9. Mass normalized quadrupole mass spectrometer (QMS) signal recorded during the heating of $Mg(B_3H_8)_2$ (left) and $Mg(B_3H_8)_2$-MgH_2 (right) with a heating ramp of 10˚C/min. The assignments of m/z=47 and 91 is hypothetical and is based on known impurities.

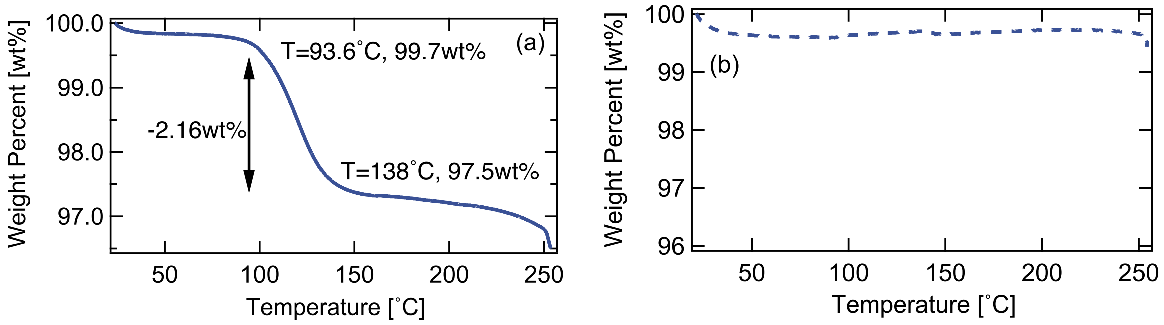

Figure S10. Thermal gravimetric analysis of $Mg(B_3H_8)_2$ (a) and $Mg(B_3H_8)_2$-MgH_2 (b) with a heating ramp of 10˚C/min. The overall weight loss for $Mg(B_3H_8)_2$-MgH_2 (b) is ~0.03wt%.

From TPD-MS of the second batch of samples, it seems like there are impurities in the samples other than $NaBPh_4$. Additionally, two unknown peaks were observed at (m/z=47) and (m/z=91) which might come from some impurities of the purchased reactants during the synthesis process. It is known that the presence of impurities from borohydride synthesis induces the formation of diborane during the decomposition of $Mg(BH_4)_2$.[4] Although there are clear differences between batch 1 and batch 2, the role of MgH_2 still holds for batch 2 that contains more impurities in the starting materials. The amount of B_2H_6 and B_5H_9 formed during the decomposition of $Mg(B_3H_8)_2$-MgH_2 is substantially reduced (~ 1 order of magnitude in intensity) compared to that of $Mg(B_3H_8)_2$. Furthermore, like demonstrated for batch 1 in **Figure 6b**, also in batch 2 is B_5H_9 released before B_2H_6. In terms of mass loss, the TPD-MS and thermal gravimetric analysis (TGA) shown in **Figure S8b** and **Figure S10b** of this second batch clearly show that the MgH_2-containing samples lose substantially less weight than the $Mg(B_3H_8)_2$ samples. The TGA was performed in a TA Instruments Q600 apparatus and Tzero Al pans with a pinhole lid.[5] To minimize the air exposure to the samples, the samples were loaded in a glovebox and quickly transferred into the TGA equipment which was continuously purged with 100 mL/min of N_2

and the measurement was started immediately thereby increasing the uncertainty on the initial weight loss values. In summary, while the synthesis protocol followed for both batches was the same, certain parameters were changed by serendipity, such as the size of the sample holder, the size of the ball milling balls, thereby drastically altering the chemical composition of the starting materials of batch 1 and batch 2. However, the results related to the role of MgH_2 were reproducible for both batches. A thermal conversion analysis of batch 2 was not perform, however, so no conclusion regarding the cyclability of samples with impurities can be drawn at this point.

S5. Quantification extracted from the XANES experiment of $Mg(B_3H_8)_2$-MgH_2

	TEY		FY	
	**Starting material**	**Heated to 100˚C**	**Starting material**	**Heated to 100˚C**
$Mg(B_3H_8)_2$	70.20	64.49	21.68	22.05
MgH_2	0	23.84	78.19	43.48
$Mg(BH_4)_2$	29.8	11.67	0	34.67

Table S2. Quantification analysis (in %) of the $Mg(B_3H_8)_2$-MgH_2 XANES data using only materials for which a standard material existed. The values are accurate within ± 3%.

S6. Molar masses used for the mass normalization of the TPD data shown in Table 4

105.3	102.89	26.32		
**$Mg(B_3H_8)_2$**	**NaBr**	**MgH_2**	**Total molecular weight**	**Scaling factor for H_2**
1	2	0	311.08	2.954
1	2	4	416.46	3.955

Table S3. Molecular weight of the different samples and the scaling factor used to normalize the data to the "active" sample mass used to extract the gravimetric capacity of H_2 from TPD shown in **Table 4**. The top row indicates the molecular weight of the different constituents of the samples investigated in this study. Because only negligeable amounts of H_2 from MgH_2 was released at $T \leq 200$°C, MgH_2 was not considered an "active" sample.

S7. Quantification obtained by ^{11}B Solid-state MAS NMR of $Mg(B_3H_8)_2$-MgH_2 under 6 bar of H_2 at 25°C before in situ variable temperature ^{11}B solid state MAS NMR experiments

**^{11}B chemical shift [ppm]**	**Assignment**	**wt%**
-32.5, -31.8, -34.1, -36.6	$B_3H_8^-$	65
-42.2	BH_4^-	11
-0.03, 17.0	BO_x	24

Table S4. Chemical composition of $Mg(B_3H_8)_2$-MgH_2 under 6 bar of H_2 obtained with ^{11}B solid-state MAS NMR at 25°C.[6]

S8. Quantification obtained from ^{11}B Solid-state MAS NMR of $Mg(B_3H_8)_2$-MgH_2 at 100°C

^{11}B chemical shift [ppm]	Assignment	wt%
-43.0, -42.1, -41.0, -39.4	BH_4^-	59
-16.0	$B_{12}H_{12}^{2-}$	0.2
-30.6, -31.8	$B_3H_8^-$	16
-13.7, -52.5	B_5H_9	4
0.01, 16.7	BO_x and B_2H_6	12

Table S5. Chemical composition of $Mg(B_3H_8)_2$-MgH_2 obtained by ^{11}B Solid-state MAS NMR at 11.7T, 5kHz MAS and 100°C.[6]

S9. Hydrogen pressure obtained for the decomposition of MgH_2

Temperature	ΔS° (J/K·mol)	Δ H° (kJ/mol)	Δ G° (kJ/mol)	p/p° (p° = 1bar)
**298**	132.35	76.17	36.7297	3.64775E-07
**300**	132.47	76.2	36.459	4.48825E-07
**400**	136.75	77.69	22.99	0.000995145
**500**	138.76	78.59	9.21	0.109109118
**600**	139.61	79.02	-4.746	2.589202857
**700**	139.69	79.1	-18.683	24.78044226
**800**	139.41	78.9	-32.628	135.0015077
**900**	139.1	78.57	-46.62	507.7960958

Table S6. Standard entropy ΔS°(J/K·mol), standard enthalpy ΔH°(kJ/mol), standard Gibbs Free Energy ΔG°(kJ/mol) and H_2 pressure for the decomposition of MgH_2 at different temperatures obtained from NIST data.[7-8]

S10. References

1.Moury, R.; Gigante, A.; Hagemann, H., An alternative approach to the synthesis of NaB_3H_8 and $Na_2B_{12}H_{12}$ for solid electrolyte applications. *Int. J. Hydrog. Energy* **2017,** *42* (35), 22417-22421.

2.Chong, M.; Matsuo, M.; Orimo, S.-i.; Autrey, T.; Jensen, C. M., Selective Reversible Hydrogenation of $Mg(B_3H_8)_2$/MgH_2 to $Mg(BH_4)_2$: Pathway to Reversible Borane-Based Hydrogen Storage? *Inorg. Chem* **2015,** *54*(8),4120-4125.

3.Gierczyk, B.; Schroeder, G.; Wojciechowski, G.; Różalski, B.; Brzezinski, B.; Zundel, G., FTIR and multinuclear magnetic resonance studies of tris(oxaalkyl) borates and their complexes with Li^+ and Na^+ cations. *Phys.Chem.Chem. Phys.***1999,***1*(20),4897-4901.

4. Stadie, N. P.; Callini, E.; Richter, B.; Jensen, T. R.; Borgschulte, A.; Züttel, A., Supercritical N_2 processing as a route to the clean dehydrogenation of porous $Mg(BH_4)_2$. *J. Am. Chem. Soc.* **2014,** *136* (23), 8181-8184.

5. Hurst, K. E.; Heben, M. J.; Blackburn, J. L.; Gennett, T.; Dillon, A. C.; Parilla, P. A., A dynamic calibration technique for temperature programmed desorption spectroscopy. *Rev. Sci. Instrum.* **2013,** *84* (2), 025103.

6. Hermanek, S., Boron-11 NMR spectra of boranes, main-group heteroboranes, and substituted derivatives. Factors influencing chemical shifts of skeletal atoms. *Chem. Rev.* **1992,** *92* (2), 325-362.

7. Maillard, R.; Sethio, D.; Hagemann, H.; Lawson Daku, L. v. M., Accurate Computational Thermodynamics Using Anharmonic Density Functional Theory Calculations: The Case Study of B–H Species. *ACS omega* **2019,** *4* (5),

8786-8794.
8. Sethio, D.; Daku, L. M. L.; Hagemann, H.; Kraka, E., Quantitative Assessment of B-B-B, B-H_b-B, and B-H_t Bonds: From BH_3 to $B_{12}H_{12}^{2-}$. *ChemPhysChem* **2019,** *20*, 1967-1977.
